# Molecular investigation of bacterial and protozoal pathogens in ticks collected from different hosts in Turkey

**DOI:** 10.1186/s13071-021-04779-2

**Published:** 2021-05-20

**Authors:** Ahmet Efe Köseoğlu, Hüseyin Can, Mervenur Güvendi, Sedef Erkunt Alak, Çağrı Kandemir, Turğay Taşkın, Samiye Demir, Gülşah Akgül, Aysu Değirmenci Döşkaya, Muhammet Karakavuk, Mert Döşkaya, Adnan Yüksel Gürüz, Cemal Ün

**Affiliations:** 1 Molecular Biology Section, Department of Biology, Faculty of Science, Ege University, Izmir, Turkey; 2 Department of Animal Science, Faculty of Agriculture, Ege University, Izmir, Turkey; 3 Zoology Section, Department of Biology, Faculty of Science, Ege University, Izmir, Turkey; 4 Department of Internal Medicine, Faculty of Veterinary Medicine, Siirt University, Siirt, Turkey; 5 Department of Parasitology, Faculty of Medicine, Ege University, Izmir, Turkey; 6 Ödemiş Technical Training College, Ege University, Izmir, Turkey

**Keywords:** *Hepatozoon canis*, *Theileria ovis*, *Babesia caballi*, *Anaplasma ovis*

## Abstract

**Background:**

The emergence of tick-borne disease is increasing because of the effects of the temperature rise driven by global warming. In Turkey, 19 pathogens transmitted by ticks to humans and animals have been reported. Based on this, this study aimed to investigate tick-borne pathogens including *Hepatozoon* spp., *Theileria* spp., *Babesia* spp., *Anaplasma* spp., *Borrelia* spp., and *Bartonella* spp. in tick samples (*n* = 110) collected from different hosts (dogs, cats, cattle, goats, sheep, and turtles) by molecular methods.

**Methods:**

To meet this objective, ticks were identified morphologically at the genus level by microscopy; after DNA isolation, each tick sample was identified at the species level using the molecular method. Involved pathogens were then investigated by PCR method.

**Results:**

Seven different tick species were identified including *Rhipicephalus sanguineus*, *R. turanicus*, *R. bursa*, *Hyalomma marginatum*, *H. anatolicum*, *H. aegyptium*, and *Haemaphysalis erinacei*. Among the analyzed ticks, *Hepatozoon* spp., *Theileria* spp., *Babesia* spp., and *Anaplasma* spp. were detected at rates of 6.36%, 16.3%, 1.81%, and 6.36%, respectively while *Borrelia* spp. and *Bartonella* spp. were not detected. *Hepatozoon* spp. was detected in *R. sanguineus* ticks while *Theileria* spp.*, Babesia* spp., and *Anaplasma* spp. were detected in *R. turanicus* and *H. marginatum*. According to the results of sequence analyses applied for pathogen positive samples, *Hepatozoon canis*, *Theileria ovis*, *Babesia caballi*, and *Anaplasma ovis* were identified.

**Conclusion:**

*Theileria ovis* and *Anaplasma ovis* were detected for the first time to our knowledge in *H. marginatum* and *R. turanicus* collected from Turkey, respectively. Also, *B. caballi* was detected for the first time to our knowledge in ticks in Turkey.

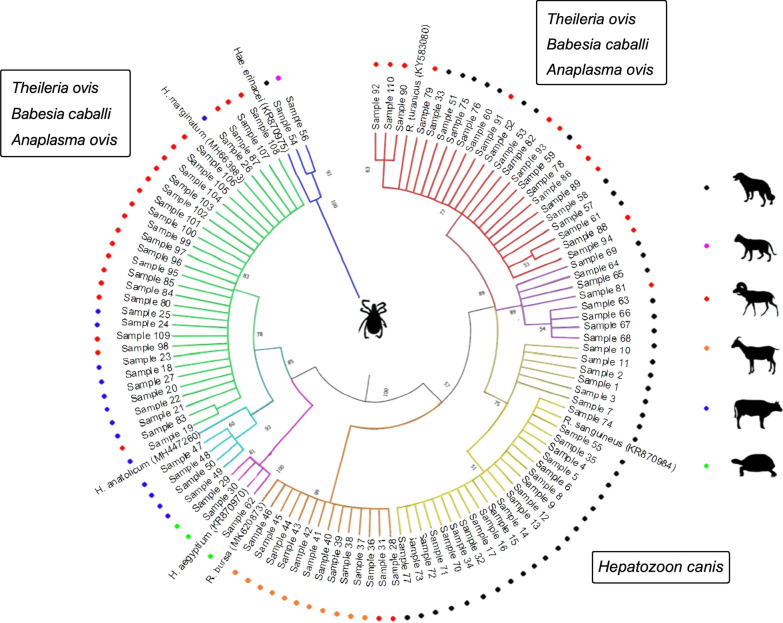

## Background

Ticks are obligate blood-sucking arachnid ectoparasites belonging to the Ixodida suborder that feeds on a wide variety of wild and domestic vertebrates excluding fish [1–3]. To date, 896 tick species identified are classified into three families: Ixodidae (hard ticks, 702 species), Argasidae (soft ticks, 193 species), and Nuttalliellidae (*Nuttalliella namaqua*, 1 species) [3]. After mosquitoes, ticks are the second most common pathogen vectors worldwide [1, 4, 5], and tick-borne diseases (TBDs) are increasingly threatening animal and human health along with causing economic losses [3, 6].

To date, 19 tick-borne pathogens have been reported in Turkey [7]. Among them, causative agents of hepatozoonosis, theileriosis, babesiosis, anaplasmosis, bartonellosis, and Lyme disease are frequently investigated in both ticks and their vertebrate hosts in Turkey and other countries. Hepatozoon, an apicomplexan intraerythrocytic genus of parasites represented by > 300 species, and belonging to the Hepatozoidae family, is commonly detected in tetrapod vertebrates and numerous hematophagous invertebrates [8, 9]. Among these species, *H. canis* and *H. felis* cause canine and feline hepatozoonosis, respectively, which are important in the veterinary field [10]. In Turkey, *H. canis* was detected in *R. sanguineus* collected from dogs and *Haemaphysalis parva* collected from red foxes [11, 12]. *Theileria* is another apicomplexan parasite that belongs to the order Piroplasmida, and it infects many domestic and wild ruminant animals. *Theileria parva*, *T. annulata*, and *T. orientalis* infect cattle [10, 13], while *T. lestoquardi*, *T. luwenshuni*, *T. separata*, and *T. ovis* infect small ruminants such as sheep and goats [14, 15]. In Turkey, *T. ovis* has been detected in *R. bursa*, *R. turanicus*, and *R. sanguineus* collected from sheep and goats [16, 17]. *Babesia* is another important apicomplexan parasite that belongs to the order Piroplasmida, which is transmitted by ticks and infects the red blood cells of various mammals such as cattle, sheep, horses, dogs, and rodents [10]. The vectors of *Babesia* parasites transmitted to humans and animals are the Ixodid tick species [18]. *Babesia caballi* together with *T. equi* causes equine piroplasmosis disease in horses and is transmitted by *Dermacentor*, *Rhipicephalus*, and *Hyalomma* [19–21]. Among bacterial pathogens, *Anaplasma* is a gram-negative bacterium that is a member of the family Anaplasmataceae in the order Rickettsiales, and it affects human and animal health by causing tick-borne diseases with species such as *A. phagocytophilum*, *A. centrale*, *A. marginale*, *A. bovis*, *A. platys*, and *A. ovis* [22–24]. Although *A. ovis* infects sheep, goats, and wild ruminants in Africa, Asia, Europe, and the US, and shows less pathogenicity than other *Anaplasma* species, it is the main species causing anaplasmosis in small ruminants with subclinical infections [25, 26]. *Borrelia* (*Borreliella*) is another bacterial pathogen in the Spirochaetaceae family that causes tick-borne Lyme borreliosis disease and is transmitted by *Ixodes* tick vectors in Europe, the Far East, and North America [27–29]. *Borrelia burgdorferi* (*s.l.*) includes 20 species, and 9 of them are known as human and animal pathogens [29]. In addition to ticks, *Bartonella*, which is another bacterial pathogen, can be transmitted by fleas and lice and can infect domestic and wild mammals, including humans. Twenty-three species have been identified, and 13 of them have been found to be associated with human diseases [30, 31]. Among *Bartonella* species, *B. henselae* and *B. clarridgeiae* cause cat-scratch disease, while *B. quintana* causes trench fever disease [31, 32]. In Turkey, *B. henselae and B. clarridgeiae* have been reported in domestic cats [33].

In this study, we aimed to investigate tick-borne pathogens including *Hepatozoon* spp., *Theileria* spp., *Babesia* spp., *Anaplasma* spp., *Borrelia* spp., and *Bartonella* spp. in tick samples collected from different hosts from the İzmir, Aydın, Şanlıurfa, and Siirt provinces of Turkey by PCR method.

## Methods

### Morphological identification of tick samples

**Fig. 1:**
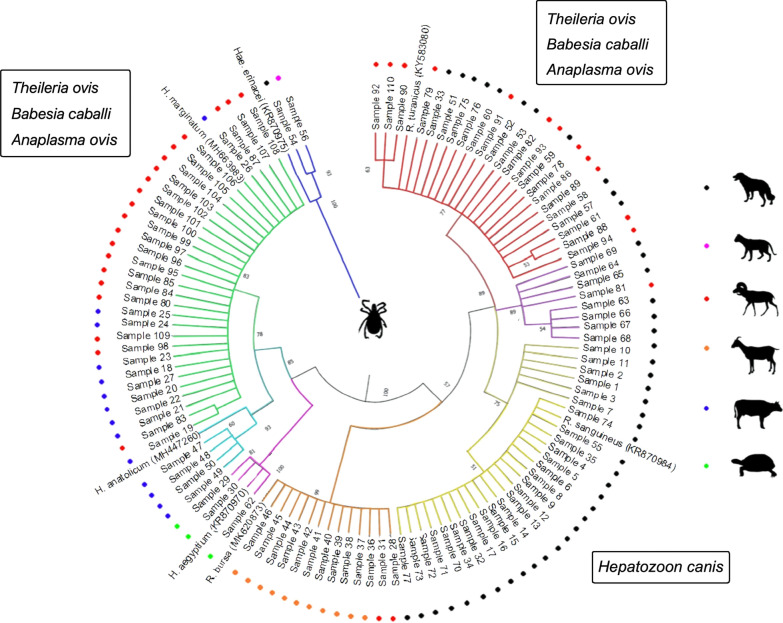
A phylogenetic tree of tick samples based on the *16S rDNA*. Each tick sample in the phylogenetic tree clustered with the sequence of the reference tick species downloaded from NCBI. Colored dots represent host organisms from which tick samples were collected. Black, pink, red, orange, blue, and green colored dots represent dog (*Canis lupus familiaris*), cat (*Felis catus*), sheep (*Ovis aries*), goat (*Capra hircus*), cattle (*Bos taurus*), and tortoise (*Testudo graeca*) hosts, respectively. Each colored branch represents a cluster of ticks in the phylogenetic tree. Pathogens are given in black boxes near the cluster of tick species in which they were detected. Silhouette images for the tick and host organisms were retrieved from PhyloPic (http://phylopic.org)

**Fig. 2:**
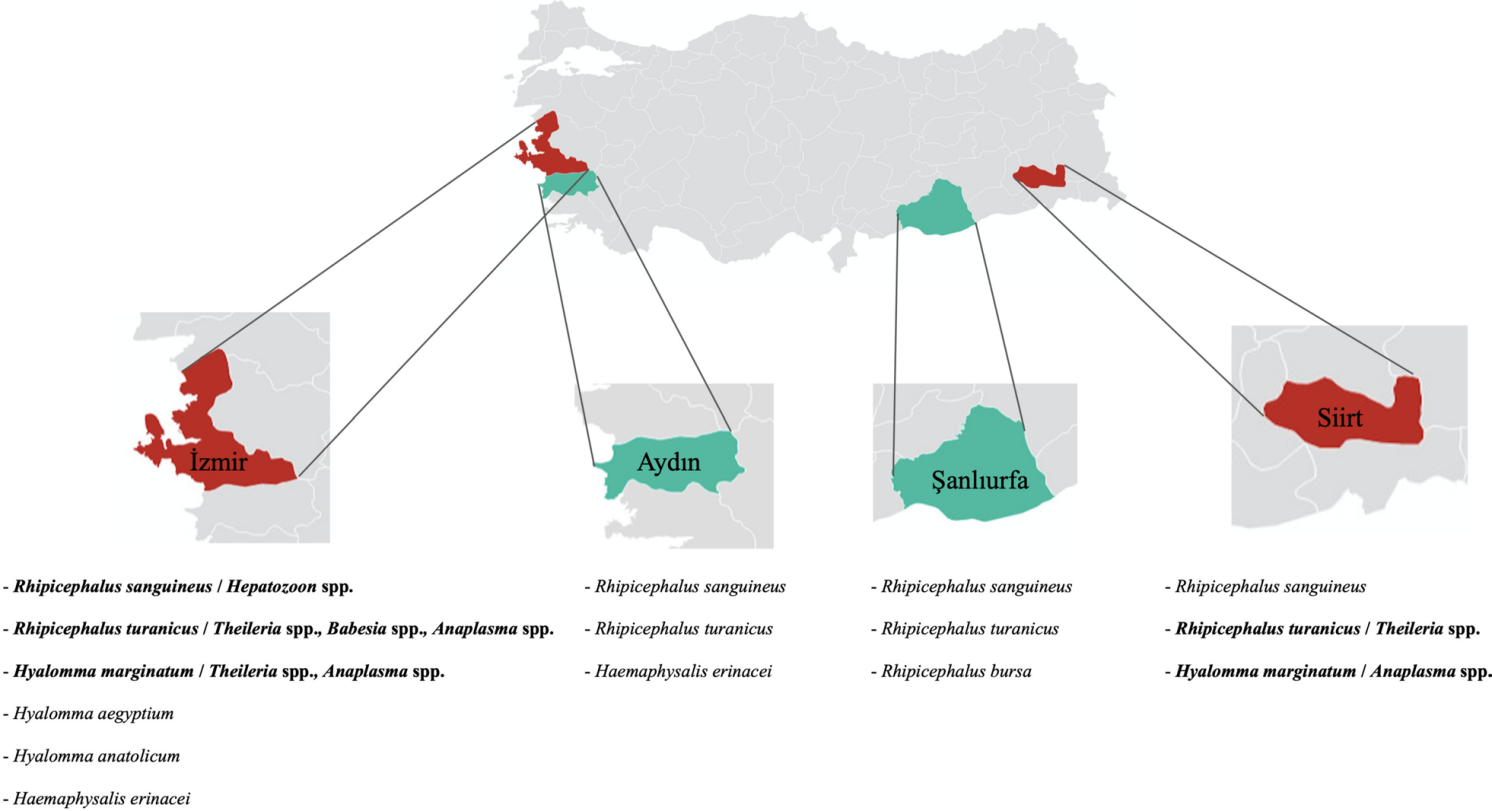
Map of Turkey showing identified tick species, pathogen DNA-positive tick species, and their collection sites. Pathogen-positive samples are in bold

Tick samples (*n* = 110) were collected from a variety of host organisms [dog (*n* = 46), cat (*n* = 1), cattle (*n* = 14), goat (*n* = 11), sheep (*n* = 35), turtles (*n* = 3)] in four provinces [İzmir (*n* = 57), Aydın (*n* = 7), Şanlıurfa (*n* = 16), Siirt = 30] of Turkey between May 2016 and July 2020 (Figs. 1 and 2). Each tick sample was collected from a different host, and all of them were selected randomly. The collections were conducted with the owners’ consent. Tick samples attached to the animals were collected, and most of them were fully engorged but some were semi-engorged. Also, all of ticks were adults except for a single tick sample that was a nymph (sample no. 62). Ticks were removed by a sterile pair of forceps and kept in separate vials filled with 70% ethanol and morphologically identified at the genus level under a stereo-microscope using identification keys as described [34–36].

### Molecular identification of tick samples

DNA was isolated from each tick sample by a commercial DNA extraction kit (Qiagen) according to the protocol of the manufacturer. Before DNA isolation, each tick sample that was kept in ethanol was rinsed twice with sterile distilled water, dried on a sterile filter, and transferred to a 1.5-ml tube containing tissue lysis buffer and proteinase K. Later, the ticks were cut in to small pieces using a sterile scalpel and homogenized by a sterile micro-pestle. The obtained homogenate was incubated at 55 °C until all small tissue pieces had been lysed, and then the DNA isolation protocol was continued.

In the molecular identification of tick samples, the mitochondrial *16S rDNA* was amplified using 5′-CCGGTCTGAACTCAGATCAAGT-3′ and 5′-CTGCTCAATGATTTTTTAAATTGCTGTGG-3 primers as described by Mendell et al. [37]. The 25 µl PCR reaction included 2 µl template DNA, 1 µl primers (0.4 mM each), 12.5 µl DreamTaq PCR Master Mix (2×, Thermo Scientific), and 8.5 µl nuclease-free water. The PCR was performed using the following protocol: 5 min initial denaturation step at 95 °C, followed by 10 cycles of 1 min at 92 °C, 1 min at 48 °C, and 90 s at 72 °C; 32 cycles of 1 min at 92 °C, 35 s at 54 °C, and 90 s at 72 °C; and a final extension of 7 min at 72 °C. All PCR products were separated in 1% agarose gel, stained with nucleic acid dye (SafeView) and visualized.

### Molecular investigation of pathogens by PCR

For detection of *Hepatozoon* spp., *Babesia*/*Theileria* spp., *Anaplasma* spp., *Borrelia* spp., and *Bartonella* spp., the used PCR methods, target genes, primers, expected PCR product sizes, and reaction and amplification conditions are shown in Table 1. For each PCR, DreamTaq PCR Master Mix (2×, Thermo Scientific) was used.

**Table 1:** PCR methods, target genes, primers, expected PCR product sizes, and reaction and amplification conditions as well as references used for detection of tick-borne pathogens

Organism	Molecular diagnostic method	Target gene	Primer	PCR product size (bp)	Reaction content	Reaction conditions	References
*Hepatozoon* spp*.*	Conventional PCR	*18S rDNA*	5′-ATACATGAGCAAAATCTCAAC-3′ 5′-CTTATTATTCCATGCTGCAG-3′	650	25 µl reaction volume (12.5 μl PCR master mix, 1 μl primers, 2 μl DNA)	95 °C 5 min, 34× (95 °C 30 s, 53 °C 30 s, 72 °C 90 s), 72 °C 5 min	[11, 55]
*Theileria* spp*.* *Babesia* spp.	Nested PCR 1	*18S rDNA*	5′-GTGAAACTGCGAATGGCTCATTAC-3′ 5-AAGTGATAAGGTTCACAAAACTTCCC-3	1609–1523	25 µl reaction volume (12.5 μl PCR master mix, 1 μl primers, 1 μl DNA)	94 °C 1 min, 30× (98 °C 10 s, 55 °C 15 s, 68 °C 45 s), 68 °C 5 min	[10]
	Nested PCR 2		5′-GGCTCATTACAACAGTTATAGTTTATTTG-3′ 5′-CGGTCCGAATAATTCACCGGAT-3′	1544–1454			
*Anaplasma* spp*.*	Nested PCR 1	*Msp4*	5′-ATGAATTACAGAGAATTGCTTGTAGG-3′ 5-TTAATTGAAAGCAAATCTTGCTCCTATG-3′	849	25 µl reaction volume (12.5 μl PCR master mix, 1 μl primers, 5 μl DNA)	94 °C 4 min, 40× (94 °C 30 s, 55 °C 30 s, 72 °C 30 s), 72 °C 10 min	[56]
	Nested PCR 2		5′-CTATTGGYGGNGCYAGAGT-3′ 5-GTTCATCGAAAATTCCGTGGTA-3′	381		94 °C 4 min, 40× (94 °C 30 s, 56 °C 30 s, 72 °C 30 s), 72 °C 10 min	
*Borrelia* spp*.*	Nested PCR 1	*OspA*	5′-CTTGAAGTTTTCAAAGAAGAT-3′ 5′-CAACTGCTGACCCCTCTAAT-3′	487	25 µl reaction volume (12.5 μl PCR master mix, 1 μl primers, 2 μl DNA)	95 °C 5 min, 40× (95 °C 15 s, 55 °C 30 s, 72 °C 45 s), 72 °C 5 min	[57]
	Nested PCR 2		5′-ACAAGAGCAGACGGAACCAG-3′ 5′-TTGGTGCCATTTGAGTCGTA-3′	350		95 °C 5 min, 40× (95 °C 15 s, 58 °C 30 s, 72 °C 45 s), 72 °C 5 min	
*Bartonella* spp*.*	Nested PCR 1	*16S-23S rDNA ITS*	5′-AAGTCGTAACAAGGT-3′ 5′-TACTGGTKCGCTATCGGTCA-3′	1800	25 µl reaction volume (12.5 μl PCR master mix, 1 μl primers, 5 μl DNA)	95 °C 5 min, 40× (94 °C 30 s, 40 °C 45 s, 72 °C 4 min), 72 °C 10 min	[58]
	Nested PCR 2		5′-TTTCTCTTTCTTCAGATGATG-3 ′5′-AAAGCAGGTGCTCTCCCAGAAAGCAGGTGCTCTCCCAG-3	587	25 µl reaction volume (12.5 μl PCR master mix, 1 μl primers, 2 μl DNA)	95 °C 5 min, 30× (94 °C 30 s, 60 °C 30 s, 72 °C 40 s), 72 °C 5 min	

### Sequencing

PCR products belonging to ticks and positive pathogen samples were sequenced, and the generated sequences were aligned and edited by MEGA7.0 software. Later, a BLAST analysis (https://blast.ncbi.nlm.nih.gov/Blast.cgi) was performed by comparing with the reference samples in National Center for Biotechnology Information (NCBI) (https://www.ncbi.nlm.nih.gov) to identify ticks and tick-borne pathogen species.

### Phylogenetic analysis

For species identification of tick samples and pathogens, phylogenetic analysis was conducted after a model test selection. Accordingly, the mitochondrial *16S rDNA* gene region (375 bp) of tick samples was aligned in MEGA7.0, and the phylogenetic tree was reconstructed by the maximum Likelihood method using the Kimura 2 (K2) model with 1000 bootstrap replications. As reference *16S rDNA* sequences, *R. sanguineus* (KR870984), *R. turanicus* (KY583080), *R. bursa* (MK620873), *H. marginatum* (MH663983), *H. anatolicum* (MH447260), *H. aegyptium* (KR870970), and *Hae. erinacei* (KR870975) deposited in NCBI database (https://www.ncbi.nlm.nih.gov) were used. The *18S rDNA* gene region (613 bp) of *H. canis* samples was aligned in MEGA7.0, and the phylogenetic tree was reconstructed by the minimum evolution method using the Tamura 3-parameter (T92) model with 1000 bootstrap replications. As reference *18S rDNA* sequences, *H. canis *(MH922768.1), *H. felis* (AB771571.1), and *Toxoplasma gondii* (L24381.1; as an outgroup) deposited in NCBI database (https://www.ncbi.nlm.nih.gov) were used. *18S rDNA* gene region (1034 bp) belonging to *T. ovis* and *B. caballi* samples was aligned in MEGA7.0, and the phylogenetic tree was reconstructed by the maximum likelihood method using the Tamura three-parameter gamma distribution (T92+G) model with 1000 bootstrap replications. As reference *18S rDNA* sequences, *T. ovis* (FJ603460.1), *T. annulata* (KF429800.1), *T. parva* (L02366.1), *B. caballi* (EU888901.1), *B. bigemina* (KM046917.1), *B. microti* (LC127372.1), and *Plasmodium falciparum* (JQ627152.1; as an outgroup) species deposited in the NCBI database (https://www.ncbi.nlm.nih.gov) were used. *Msp4* gene sequence (321 bp) of *A. ovis* sample was aligned with reference sequences, and a phylogenetic tree was reconstructed by the maximum likelihood method using the Kimura-2 (K2) model with 1000 bootstrap replications. As reference *Msp4* sequences, *A. ovis* (KU497712.1), *A. phagocytophilum* (KC847317.1), and *A. marginale* (KU497715.1) species deposited in NCBI database (https://www.ncbi.nlm.nih.gov) were used.

### Statistical analysis

Molecular detection proportions between the Aegean region and Southeastern Anatolia region as well as between tick species were computed, and comparison of the proportions was performed by the chi-square test using the PASW Statistics 18 software. Statistically significant differences were determined at *P* < 0.05.

## Results

### Morphological and molecular identification of ticks

Among 110 tick samples, morphological analysis showed that there were three genera including *Rhipicephalus* (*n* = 71; 64.5%), *Hyalomma* (*n* = 37; *n* = 33.6%), and *Haemaphysalis* (*n* = 2; 1.8%). According to NCBI-BLAST and phylogenetic analysis results, *R. sanguineus* (*n* = 27; 24.5%), *R. turanicus* (*n* = 31; 28.1%), *R. bursa* (*n* = 13; 11.8%), *H. marginatum* (*n* = 30; 27.2%), *H. anatolicum* (*n* = 4; 3.6%), *H. aegyptium* (*n* = 3; 2.72%), and *Hae. erinacei* (*n* = 2; 1.8%) were identified (Fig. 1).

### Molecular detection rates of involved pathogens

At least one pathogen DNA was amplified from 31 of 110 (28.1%) tick samples studied. Three tick samples (3/110; 2.72%) including *R. turanicus* and *H. marginatum* species were detected to carry DNA of at least two different pathogens. Tick species that were detected to harbor the pathogen DNA were *R. turanicus, H. marginatum,* and *R. sanguineus*. The detection rates of pathogen DNA were 25.9% (7/27), 32.2% (10/31), and 46.6% (14/30) for *R. sanguineus, R. turanicus,* and *H. marginatum,* respectively. *Hepatozoon* spp. was detected in *R. sanguineus* collected from dogs in İzmir, and the detection rate of *Hepatozoon* spp. DNA was 6.36% (7/110). Six of them were successfully sequenced and identified as *H. canis* (Table 2). *Theileria* spp. was detected in 18 tick samples whereas *Babesia* spp. in two tick samples. DNA detection rates were 16.3% (18/110) and 1.81% (2/110) for *Theileria* spp. and *Babesia* spp., respectively. Both were detected in *R. turanicus* and *H. marginatum* collected from sheep (Table 2). *Babesia* spp. was found in tick samples collected from İzmir whereas *Theileria* spp. in İzmir as well as Siirt. Among these positive samples, 15 samples were successfully sequenced, and 13 of them were identified as *T. ovis*, while 2 were identified as *B. caballi*. *Anaplasma* spp*.* were detected in seven tick samples including *H. marginatum* and *R. turanicus* collected from sheep in İzmir and Siirt. The detection rate of *Anaplasma* spp. DNA was 6.36% (7/110) in tick samples analyzed. Among positive samples, a single positive sample detected in *R. turanicus* was successfully sequenced and identified as *A. ovis* (Table 2). *Borrelia* spp. and *Bartonella* spp. were not detected among tick samples analyzed.

**Table 2:** The detected tick-borne pathogen species along with tick species, host organism, locality, detection method, percentage of nucleotide identity, and molecular detection rate

Pathogen species	Sample no.	Tick species	Locality	Host organism	Molecular diagnostic method	Accession number, percentage of nucleotide identity	Molecular detection rate
*Hepatozoon canis*	3	*Rh. sanguineus*	İzmir	Dog	Conventional PCR	LC428208.1; 100%	6.36% (7/110)
*Hepatozoon canis*	4	*Rh. sanguineus*	İzmir	Dog	Conventional PCR	MH615006.1; 99%	
*Hepatozoon canis*	6	*Rh. sanguineus*	İzmir	Dog	Conventional PCR	LC018209.1; 99%	
*Hepatozoon canis*	7	*Rh. sanguineus*	İzmir	Dog	Conventional PCR	LC018209.1; 100%	
*Hepatozoon canis*	11	*Rh. sanguineus*	İzmir	Dog	Conventional PCR	LC018209.1; 100%	
*Hepatozoon canis*	14	*Rh. sanguineus*	İzmir	Dog	Conventional PCR	LC428208.1; 100%	
*Hepatozoon* spp*.*	15	*Rh. sanguineus*	İzmir	Dog	Conventional PCR	^a^	
*Theileria ovis*	78	*Rh. turanicus*	Siirt	Sheep	Nested PCR	MN493111.1; 100%	16.3% (18/110)
*Theileria ovis*	79	*Rh. turanicus*	Siirt	Sheep	Nested PCR	MN493111.1; 100%	
*Theileria ovis*	85	*Rh. turanicus*	Siirt	Sheep	Nested PCR	MN493111.1; 100%	
*Theileria ovis*	88	*Rh. turanicus*	Siirt	Sheep	Nested PCR	MN493111.1; 100%	
*Theileria ovis*	89	*Rh. turanicus*	Siirt	Sheep	Nested PCR	MN493111.1; 100%	
*Theileria ovis*	90	*Rh. turanicus*	Siirt	Sheep	Nested PCR	MN493111.1; 100%	
*Theileria ovis*	91	*Rh. turanicus*	Siirt	Sheep	Nested PCR	MN493111.1; 100%	
*Theileria ovis*	92	*Rh. turanicus*	Siirt	Sheep	Nested PCR	MN493111.1; 100%	
*Theileria ovis*	94	*Rh. turanicus*	İzmir	Sheep	Nested PCR	MN493111.1; 100%	
*Theileria* spp*.*	95	*Hy. marginatum*	İzmir	Sheep	Nested PCR	^a^	
*Theileria ovis*	97	*Hy. marginatum*	İzmir	Sheep	Nested PCR	MN493111.1; 100%	
*Theileria ovis*	99	*Hy. marginatum*	İzmir	Sheep	Nested PCR	MN493111.1; 100%	
*Theileria* spp*.*	100	*Hy. marginatum*	İzmir	Sheep	Nested PCR	^a^	
*Theileria ovis*	101	*Hy. marginatum*	İzmir	Sheep	Nested PCR	MN493111.1; 99.9%	
*Theileria* spp*.*	103	*Hy. marginatum*	İzmir	Sheep	Nested PCR	^a^	
*Theileria* spp*.*	104	*Hy. marginatum*	İzmir	Sheep	Nested PCR	^a^	
*Theileria ovis*	105	*Hy. marginatum*	İzmir	Sheep	Nested PCR	MN493111.1; 100%	
*Theileria* spp*.*	107	*Hy. marginatum*	İzmir	Sheep	Nested PCR	^a^	
*Babesia caballi*	109	*Hy. marginatum*	İzmir	Sheep	Nested PCR	MN629354.1; 100%	1.81% (2/110)
*Babesia caballi*	110	*Rh. turanicus*	İzmir	Sheep	Nested PCR	MN629354.1; 100%	
*Anaplasma* spp*.*	83	*Hy. marginatum*	Siirt	Sheep	Nested PCR	^a^	6.36% (7/110)
*Anaplasma* spp*.*	84	*Hy. marginatum*	Siirt	Sheep	Nested PCR	^a^	
*Anaplasma* spp*.*	86	*Hy. marginatum*	Siirt	Sheep	Nested PCR	^a^	
*Anaplasma ovis*	94	*Rh. turanicus*	İzmir	Sheep	Nested PCR	MN307492.1; 99.67%	
*Anaplasma* spp*.*	97	*Hy. marginatum*	İzmir	Sheep	Nested PCR	^a^	
*Anaplasma* spp*.*	98	*Hy. marginatum*	İzmir	Sheep	Nested PCR	^a^	
*Anaplasma* spp*.*	99	*Hy. marginatum*	İzmir	Sheep	Nested PCR	^a^	

^a^Indicates that sequence data were not obtained

The percent identity rate among *H. canis* isolates varied from 99.05 to 100%, whereas for *T. ovis* isolates, it varied from 99.9 to 100%. The percent identity rate was 100% for *B. caballi* isolates.

The pathogen DNA positivity proportion detected in the Aegean region was statistically significantly higher than in the Southeastern Anatolia region (*P* < 0.05). Also, no statistically significant relationship was found in terms of the pathogen detection proportion among pathogen-positive tick species (*P* > 0.05).

### Phylogenetic trees

**Fig. 3:**
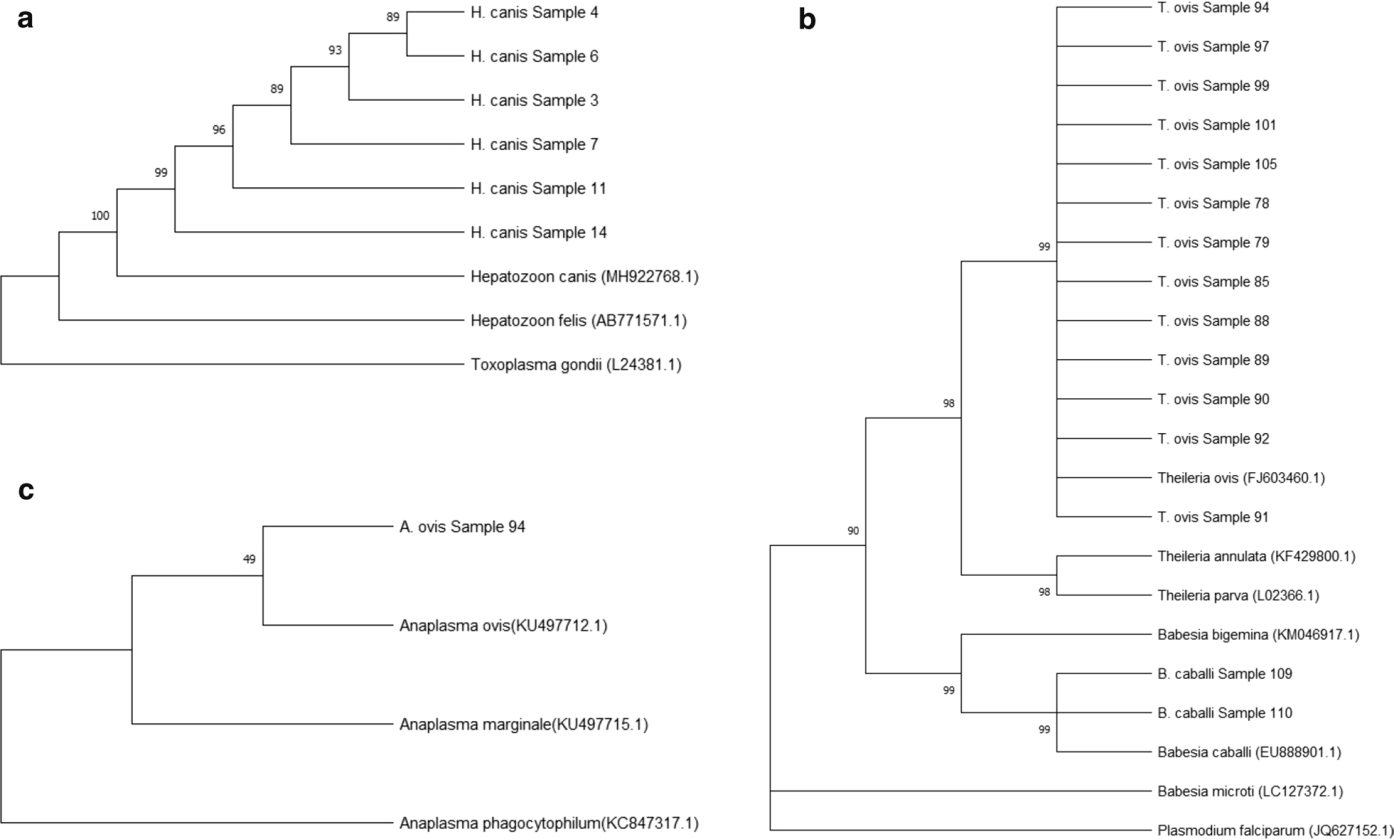
Phylogenetic trees of tick-borne pathogens sequenced in this study. **a** *Hepatozoon canis 18S rDNA* phylogenetic tree; **b** *Theileria ovis* and *Babesia caballi 18S rDNA* phylogenetic tree; **c** *Anaplasma ovis Msp4* phylogenetic tree

All analyzed species clustered with their own reference sample, and unexpected branches containing different tick species were not observed in the phylogenetic tree (Fig. 1). In addition, although eight tick sequences (samples 63, 64, 65, 66, 67, 68, 69, and 81) clustered in the same proximity to both *R. turanicus* and *R. sanguineus* reference species in the phylogenetic tree (Fig. 1), these sequences matched with *R. turanicus* in the BLAST analysis. Similarly, unexpected or mixed branches containing different species were not observed in phylogenetic trees constructed for each pathogen (Fig. 3).

## Discussion

The number of ticks has increased in suitable ecological habitats because of the rising temperature driven by global warming. To date, 19 tick-borne pathogens were reported in Turkey [7]. Although prevalence studies conducted for tick-borne pathogens are present in Turkey (Table 3), the number of these studies is insufficient in the Aegean region of Turkey, and thus the prevalence of tick-borne pathogens is incompletely known in this region. The present study aimed to investigate the important tick-borne pathogens in tick samples collected from four different provinces of Turkey especially involving the Aegean region. For this aim, identification of tick samples was conducted and *R. sanguineus* (24.5%), *R. turanicus* (28.1%), and *H. marginatum* (27.2%) were confirmed as the most prevalent tick species. Previous studies conducted in Turkey demonstrated that *R. turanicus* and *H. marginatum* were prevalent, in line with our study except for *R. sanguineus* [38, 39].

**Table 3:** Molecular prevalence of *Hepatozoon canis*, *Theileria ovis*, and *Anaplasma ovis*, detected in previous studies, in ticks from Turkey

Pathogen species	Locality	Host organism	Tick species	Diagnostic method	Prevalence	References
*Hepatozoon canis*	Diyarbakır	Dog	*Rh. sanguineus*	Conventional PCR	20.58% (14/68 pool)	[11]
	Ankara	Red fox	*Hae. parva*	Conventional PCR	33.3% (1/3 pool)	[12]
*Theileria ovis*	Elazığ	Sheep, Goat	*Rh. bursa*	Conventional PCR	19.27% (37/192 individual)	[16]
	Bolu, Kastamonu, Çorum, Samsun, Tokat, Giresun, Bayburt	Sheep, Goat	*Rh. bursa*	Conventional PCR	2.37% (10/49 pool)	[17]
		Sheep, Goat	*Rh. turanicus*	Conventional PCR	2.27% (13/70 pool)	[17]
		Sheep, Goat	*Rh. sanguineus*	Conventional PCR	1.47% (1/6 pool)	[17]
*Anaplasma ovis*	Ankara	Boar, Rabbit, Fox	*Rh. turanicus, De. marginatus*, *Hae. parva*, *Hy.* spp*.*, *Hy. marginatum*, *Hy. excavatum*, *Hy. aegyptium*	Conventional PCR	0% (0/445 individual and 0/102 pool)	[49]
	Ankara, Bolu, Kırşehir	Cattle, Sheep, Goat, Dog	*Rh. sanguineus*, *Rh. turanicus*, *Rh. bursa*, *De. marginatus*, *Hy. marginatum*, *Hy. excavatum*, *Hy. anatolicum*, *Hae. parva*, *Hae. inermis*, *Ix. ricinus*	Conventional PCR	0% (0/75 adult female individual and 0/151 larval pool)	[50]
	Ankara	Sheep	*Rh. sanguineus*	Conventional PCR	0.82% (2/242 individual)	[51]

In addition, such important protozoan and bacterial pathogens also have been investigated in this study; 35.9% of tick samples collected from the Aegean region were pathogen DNA-positive whereas 23.9% of ticks collected from the Southeastern Anatolia region were pathogen DNA-positive. *Hepatozoon* spp. was detected by conventional PCR with a rate of 6.36% in *R. sanguineus* collected from dogs in İzmir province of Turkey and identified as *H. canis* (Table 2). Although *R. sanguineus* is known to be the main vector for *H. canis*, the vectoring possibilities of other tick species including *R. turanicus*, *Hae. longicornis*, *Hae. flava*, and *Amblyomma ovale* have been reported [40–42]. In Turkey, *H. canis* was detected by conventional PCR with a prevalence of 33.3% in *Hae. parva* collected from red foxes in Ankara while it was detected by conventional PCR with a prevalence of 20.58% in *R. sanguineus* collected from dogs in Diyarbakır [11, 12] (Table 3). In addition to Turkey, *H. canis* was detected by conventional PCR in adult *R. sanguineus* species and nymph *Ixodes ricinus* collected from dogs in Italy with prevalences of 33% and 5%, respectively [43]. These findings show that *Hae. parva* also can harbor the *H. canis* in Turkey in addition to the main vector, *R. sanguineus*.

In this study, *Theileria* spp. was detected by nested PCR with a rate of 16.3% in *R. turanicus* and *H. marginatum* collected from sheep in İzmir and Siirt provinces and identified as *T. ovis* (Table 2). Also, the present study is the first reporting the detection of *T. ovis* in *H. marginatum* in addition to *R. bursa, R. turanicus*, and *R. sanguineus* in Turkey. For example, *T. ovis* was detected by conventional PCR with a prevalence of 19.27% in *R. bursa* collected from sheep and goats in Elazig province of Turkey [16] (Table 3), and it was known that *T. ovis* was the most common *Theileria* species in small ruminants and its vector was *R. bursa* in the Mediterranean basin and Turkey [14, 16, 44, 45]. However, another work conducted in the Black Sea region of Turkey (Bolu, Kastamonu, Çorum, Samsun, Tokat, Giresun, and Bayburt) detected *T. ovis* by conventional PCR in *R. turanicus* and *R. sanguineus* in addition to *R. bursa* collected from sheep and goats with prevalences of 2.27%, 1.47%, and 2.37%, respectively [17] (Table 3).

In this study, *Babesia* spp. was detected by nested PCR with a rate of 1.81% in *H. marginatum* and *R. turanicus* collected from sheep in İzmir and identified as *B. caballi*. This was the second important first report in this study, demonstrating the presence of *B. caballi* in two different tick species in Turkey (Table 2). A previous study conducted in Erzurum province of Turkey screened *B. caballi* by multiplex PCR in horses but was not detected [20] while another study detected it with a prevalence of 3% by PCR in horses in Ankara [46]. Also, no report has detected *B. caballi* in ticks in Turkey. Apart from Turkey, *B. caballi* was detected by conventional PCR with a prevalence of 7.6% in *R. evertsi evertsi* from horses and donkeys in Nigeria while it was detected by nested PCR with a prevalence of 12.9% in *Dermacentor nuttalli* from horses in Mongolia [47, 48]. Also, *B. caballi* was detected with a prevalence of 14.55% in 2017 while it was detected with a prevalence of 27.59% in 2018 by real-time PCR in *R. bursa* collected from vegetation in Italy [21].

In this study, *Anaplasma* spp. was detected by nested PCR with a rate of 6.36% in *H. marginatum* and *R. turanicus* collected from sheep in the Siirt and İzmir provinces of Turkey, and only one sample detected in *R. turanicus* could be sequenced and identified as *A. ovis* (Table 2). This was the third important first report in this study demonstrating the presence of *A. ovis* in *R. turanicus* in Turkey (Table 2). In Turkey, *A. ovis* was not detected by conventional PCR in ticks collected from several animals in Ankara [49] (Table 3). Similarly, a previous work comprising Ankara, Bolu, and Kırşehir provinces did not detect *A. ovis* by conventional PCR in several ticks collected from animals including cattle, sheep, goats, and dogs [50] (Table 3). However, another study detected *A. ovis* by PCR with a prevalence of 0.82 (2/242) in *R. sanguineus* collected from sheep in Ankara [51] (Table 3). Apart from Turkey, *A. ovis* was detected by conventional PCR with a prevalence of 2.3% in *Hae. longicornis* collected from sheep in China while it was detected by conventional PCR with a prevalence of 2.4% in *D. nuttalli* collected from sheep and goats in Mongolia [23, 52]. Also, *A. ovis* was detected by real-time PCR with a prevalence of 20.3% in *R. bursa* collected from goats in France [26].

Interestingly, *Borrelia* spp. and *Bartonella* spp. were not detected among tick samples analyzed in the present study. Indeed, *Borrelia* spp. has been detected at high prevalence rates in tick samples analyzed in previous studies conducted in Turkey. For example, *Borrelia* spp. was detected by nested PCR with a prevalence of 20% in *R. turanicus* collected from boars in Ankara [49]. A different study conducted in İstanbul detected *Borrelia* spp. by conventional PCR with prevalence rates of 44% and 39% in adult and nymph forms of *H. aegyptium*, respectively, which were collected from tortoises (*Testudo graeca*) [53]. Also, *B. burgdorferi* was detected by nested PCR with prevalence rates of 38.7% and 11.4% in İstanbul and Kırklareli provinces, respectively [54]. In addition, to date, the molecular prevalence of *B. henselae* has only been reported in domestic cats in Turkey [7, 33], and no molecular prevalence study is available on the existence of *Bartonella* spp. in ticks.

This study has some limitations. First is the small sample size. A more comprehensive study with more samples can enable reaching a more precise pathogen detection rate as well as detect the *Borrelia* spp. and *Bartonella* spp. in these study areas. The other is the use of a single molecular diagnostic method. In addition to this method, the use of microscopy and culture methods or additional molecular methods such as RNA analyses indicating pathogen-related gene expression can indicate that ticks were infected with the detected pathogens and these pathogens could be transmitted by ticks to vertebrate hosts.

## Conclusions

*Hepatozoon canis, T. ovis*, *B. caballi*, and *A. ovis* were detected among tick samples studied contrary to *Borrelia* spp. and *Bartonella* spp. Overall, at least one pathogen DNA was detected in 28.1% of tick samples studied. Among these findings, three of them are of great importance for studies conducted in this field in Turkey. The first one is the first detection of *T. ovis* in *H. marginatum* whereas the second one is the first detection of *A. ovis* in *Rh. turanicus,* and the third one is the first detection of *B. caballi* in both *H. marginatum* and *R. turanicus* species, which have veterinary and medical importance*.*

## Data Availability

All sequences obtained from pathogens were deposited into GenBank (National Center for Biotechnology Information Search database). Provided GenBank Accession numbers are as follows: MW810676, MW810677, MW810626, MW810627, MW810628, MW810629, MW810630, MW810631, MW810474, MW810475, MW810476, MW810477, MW810478, MW810479, MW810480, MW810481, MW810482, MW810483, MW810484, MW810485, MW810486, and MW821793.
